# *Infectious Diseases of Poverty* reviewer acknowledgement 2015

**DOI:** 10.1186/s40249-016-0114-0

**Published:** 2016-03-17

**Authors:** Xiao-Nong Zhou

**Affiliations:** National Institute of parasitic Diseases, Shanghai, China CDC, China

## Abstract

The editors of *Infectious Diseases of Poverty* would like to thank all our reviewers who have contributed to the journal in volume 4 (2015).

**Hanin Abdel-Haq**

Italy

**Suneth Agampodi**

Sri Lanka

**Fiacre A.R. Agossa**

France

**Valéria Magalhães Aguiar**

Brazil

**Maryam Alavi**

Australia

**Nahid Ali**

India

**Shahzad Ali**

Pakistan

**Julio Aliberti**

United States of America

**Salem Alkoshi**

Malaysia

**Jaffar Al-Tawfiq**

Saudi Arabia

**Frederico Figueiredo Amâncio**

Brazil

**Fentie Ambaw**

United Kingdom

**Stephen Ambu**

Malaysia

**Zuhair Amr**

Jordan

**SS Andreadis**

Greece

**Andrea Angheben**

Italy

**Gloria Ansa**

Ghana

**Maria Antoniou**

Greece

**Ahmed Arshad**

Pakistan

**G. V. Asokan**

Bahrain

**Justine Assenga**

Tanzania, United Republic Of

**Salah Awaidy**

Oman

**James Ayukepi Ayukekbong**

Sweden

**Santi P Sinha Babu**

India

**Subash Babu**

India

**David Barbosa**

Brazil

**Sukhadeo Barbuddhe**

India

**Amy Barrette**

Switzerland

**Devra Barter**

United States of America

**Leonardo Basco**

France

**Vagner Wilian Batista**

Brazil

**Soeren Becker**

Germany

**Tatiana Belkina**

Czech Republic

**Yunus E. Beyhan**

Turkey

**Andrea K Blanchard**

Canada

**David Blok**

Netherlands

**Boakye Boatin**

Ghana

**Monika Bociaga-Jasik**

Poland

**Maha Bouzid**

United Kingdom

**Barry M. Bradford**

United Kingdom

**Andreia F Brilhante**

Brazil

**Christine Budke**

United States of America

**Jesse Bump**

United States of America

**Stephanie Burniston**

United Kingdom

**Jun Cao**

China

**Helene Carabin**

United States of America

**Luís Cardoso**

United States of America

**Orlando Cenciarelli**

Italy

**Kwang Poo Chang**

United States of America

**Himanshu Chaturvedi**

India

**Jun-Hu Chen**

China

**Gerardo Chowell**

Georgia

**Luc Coffeng**

Netherlands

**Ulisses Confalonieri**

Brazil

**Ian Gillespie Cook**

United Kingdom

**James A. Cotton**

Canada

**Janet Cox-Singh**

United Kingdom

**Zulma Cucunubá**

Colombia

**Sabyasachi Das**

India

**Emmie de Wit**

United States of America

**Nana Rose Diakite**

Cote D’Ivoire

**João Carlos Pinto Dias**

Brazil

**Emilio Dirlikov**

Canada

**Hui-Fen Dong**

China

**Yan Du**

China

**Bereket Duko**

Ethiopia

**Karthickeyan Duraisamy**

India

**Valentina Virginia Ebani**

Italy

**Yehenew Ebstie**

Ethiopia

**Akinwale Efunshile**

Denmark

**Grace Egeland**

Norway

**Uwem Ekpo**

Nigeria

**Mark Erfe**

United States of America

**Bruno Eto**

France

**Shirzad Fallahi**

Iran, Islamic Republic Of

**Ann Fallon**

United States of America

**Zhen-Chuan Fan**

China

**Chia-Kwung Fan**

Taiwan, China

**Li-Qun Fang**

China

**Jun Feng**

China

**Magilé C. Fonseca**

Cuba

**Florence Fouque**

Swaziland

**Jana Fried**

United Kingdom

**Ricardo Fujiwara**

Brazil

**Francesco Maria Fusco**

Italy

**Luis-Alberto Gaitán-Cepeda**

Mexico

**Shu-Jing Gao**

China

**Amadou Garba**

Switzerland

**Andrés Garchitorena**

United States of America

**Melissa N. Garcia**

United States of America

**Giuliano Garofolo**

Italy

**Mohammadreza Gholami**

Iran, Islamic Republic Of

**Luis Miguel González**

Spain

**Catherine Gordon**

Australia

**Rajeev Goyal**

India

**Carlos Graeff-Teixeira**

Brazil

**Jack ET Grimes**

United Kingdom

**Gigi Gronvall**

United States of America

**Ashley Grosso**

United States of America

**Rodrigo Guabiraba**

France

**Wei Guo**

China

**Narayan Gyawali**

Australia

**Fisaha Haile**

Ethiopia

**Yara Halasa**

United States of America

**Nguyen B Hoa**

Viet Nam

**Sung-Tae Hong**

Korea, Republic Of

**Wei Hu**

China

**Felicien Ilunga-Ilunga**

Congo

**Asma Iqbal**

Canada

**Laura F. Jefferys**

Germany

**Julie Jesson**

France

**Min-Jun Ji**

China

**Feng-Liang Jin**

China

**Maria Vang Johansen**

Denmark

**Roch Christian Johnson**

Benin

**Nicholas Johnson**

United Kingdom

**Seth Judson**

United States of America

**Mamadou Kaba**

South Africa

**George Kafatos**

United Kingdom

**Ajeet Kaushik**

United States of America

**Faiz Kermani**

France

**Thomas Kesteman**

Madagascar

**Samson Kiware**

Tanzania

**Stefanie Knopp**

Switzerland

**Ismail Soner Koltas**

Turkey

**Randall Kramer**

United States of America

**Stefanie Krauth**

Chile

**Anuja Krishnan**

United States of America

**Karen Krogfelt**

Denmark

**Annette Kuesel**

Switzerland

**Agnes Kurniawan**

Indonesia

**Kang Yiu Lai**

Hong Kong, China

**Moses Laman**

Papua New Guinea

**Essoya D Landoh**

Togo

**Samia Laokri**

Belgium

**David Larsen**

United States of America

**Anne Laudisoit**

United Kingdom

**Jae Hoon Lee**

Korea, Democratic People’S Republic Of

**Marilena Leis**

Italy

**Shi-Zhu Li**

China

**Ying Li**

China

**Xin-Xu Li**

China

**Chang-Wei Li**

United States of America

**Xiang-Rui Li**

China

**Ie-Bin Lian**

Taiwan, China

**Marshall Lightowlers**

Australia

**Wen Lin**

China

**Ji-Ming Liu**

Hong Kong, China

**Lu Liu**

China

**Ming-Yuan Liu**

China

**Eric S Loker**

United States of America

**Knut Lönnroth**

Switzerland

**Anna Lena Lopez**

Philippines

**Da-Bing Lu**

China

**Zhao-Rong Lun**

China

**Shan Lv**

China

**Ye Ma**

China

**Ya-Jun Ma**

China

**Gino C. Matibag**

Philippines

**Gerald Amandu Matua**

Oman

**Rooyen Mavenyengwa**

Zimbabwe

**Webster Mavhu**

Zimbabwe

**Don McManus**

Australia

**KE McMillan**

Australia

**Takafira Mduluza**

Zimbabwe

**Ann Miller**

United States of America

**J. Michael Miller**

United States of America

**Oriol Mitjà**

Papua New Guinea

**David Molyneux**

United Kingdom

**Estrella Montero**

Spain

**Catrin Moore**

United Kingdom

**Filbert Mpogoro**

Tanzania

**Ivo Mueller**

Spain

**Martin W Mutuku**

Kenya

**Adamson Muula**

Malawi

**Ramesh Nagarajappa**

India

**Pradeep Nair**

India

**Mercy Nassali**

Bangladesh

**Ignatius C Ndong**

Cameroon

**Jintana Ngamvithayapong-Yanai**

Thailand

**Abel Ntambue**

Congo, The Democratic Republic Of The

**Ndudim Ogo**

United States of America

**Omoyemi Ogwumike**

Nigeria

**Olusola Ojurongbe**

Nigeria

**Anna L. Okello**

United Kingdom

**Joseli Oliveira-Ferreira**

Brazil

**Samuel Anu Olowookere**

Nigeria

**Nyamongo Onkoba**

Kenya

**Anyebe Onoja**

Nigeria

**Gabriella Ortore**

Italy

**Trevino Pakasi**

Indonesia

**Jun-Xiong Pang**

Singapore

**Andrea Pantoja**

Swaziland

**Daniel M. Parker**

Thailand

**John Pasick**

Canada

**Jorge Pedrosa**

Portugal

**Adalberto Pereira Filho**

Brazil

**Lucia Monserrat Pérez-Navarro**

Mexico

**Guey Chuen Perng**

Taiwan, China

**Punnee Pitisuttithum**

Thailand

**Ranjan Premaratna**

Sri Lanka

**Xiao-Peng Qi**

China

**Men-Bao Qian**

China

**Zhi-Jiang Qin**

China

**Soheila Rabiepoor**

Iran, Islamic Republic Of

**Senaka Rajapakse**

Sri Lanka

**C.P. Ramachandran**

Malaysia

**José Manuel Ramos**

Ethiopia

**Ram Rangsin**

Thailand

**Felipe Dutra Rêgo**

Brazil

**Hong-Yan Ren**

China

**Blanca Restrepo**

United States of America

**Nicolás Robinson-García**

Spain

**Minakshi Rohilla**

India

**Gianluigi Rossi**

Italy

**Xavier Roura**

Brazil

**Somenath Roy**

India

**Nicole Rübsamen**

Germany

**Charles Rupprecht**

United States of America

**Maren Johanne Heilskov Rytter**

Denmark

**Moussa Sacko**

Mali

**Evans L Sagwa**

South Africa

**Ashis Saha**

India

**Rehana Salam**

Pakistan

**Helena Lúcia Carneiro Santos**

Brazil

**Ari Winasti Satyagraha**

Indonesia

**Matthias Schmitz**

Germany

**Poongulali Selvamuthu**

India

**Emmanuel Serrano**

Portugal

**Lillian Seu**

Zambia

**Heba Shawky**

Egypt

**Yu-Juan Shen**

China

**Ben-Yun Shi**

China

**Ambuj Shrivastava**

India

**Denise Silva**

Brazil

**Cheolho Sim**

United States of America

**Sarah Simmons**

United States of America

**B.B. Singh**

India

**Susanne H. Sokolow**

United States of America

**Maja Stanojevic**

Serbia And Montenegro

**Michelle Stanton**

United Kingdom

**Peter Steinmann**

Switzerland

**Xin-Zhuan Su**

United States of America

**Xiao-You Su**

China

**Bindu Sukumaran**

Singapore

**Irene Sumbele**

Cameroon

**Claudette Simone Sutherland**

Switzerland

**Hiroshi Suzuki**

Japan

**Annika C. Sweetland**

Colombia

**Katsushi Tajima**

Japan

**Hiko Tamashiro**

Japan

**Ernest Tambo**

South Africa

**Chee Wah Tan**

Malaysia

**Biruhalem Taye**

Ethiopia

**Walter R. J. Taylor**

Switzerland

**Khin Thet Wai**

Myanmar

**Roger Thomas**

Canada

**R.C. Andrew Thompson**

Australia

**Kamala Thriemer**

Belgium

**Lucas Tirloni**

Brazil

**Mohamad-Ali Trad**

France

**Tiziano Tuccinardi**

Italy

**Claire M Tully**

United Kingdom

**Begna Tulu**

Ethiopia

**Kingsley Ukwaja**

Nigeria

**Ravi Kant Upadhyay**

India

**Maria Isabel Veiga**

Sweden

**Raman Velayudhan**

Switzerland

**Patrícia Sampaio Tavares**

Brazil

**Elvina Viennet**

Australia

**Maria Vitale**

Italy

**Tyson Volkmann**

United States of America

**Dominique Angèle Vuitton**

France

**Anjuli Wagner**

United States of America

**Wei Wang**

China

**Yong Wang**

China

**Shan-Qing Wang**

China

**Ming-Gui Wang**

China

**Qin Wang**

China

**Kinley Wangdi**

Australia

**David Weetman**

United Kingdom

**Xiao-Lin Wei**

China

**Mitchell Weiss**

Switzerland

**Thomas Weitzel**

Germany

**Yu-Feng Wen**

China

**Timothy Eoin West**

United States of America

**Bruce Wilcox**

Thailand

**La’Marcus Wingate**

United States of America

**Viroj Wiwanitkit**

Thailand

**Wendy Wong**

Hong Kong, China

**Shou-Li Wu**

China

**Wolfgang Kratzer**

Germany

**Shang Xia**

China

**Ning Xiao**

China

**Jian-Nong Xu**

United States of America

**Jing Xu**

China

**Kuang-Yao Yang**

Taiwan, China

**Kun Yang**

China

**Guo-Jing Yang**

China

**Peiling Yap**

Switzerland

**Hosup Yoon**

Singapore

**Chee-Fu Yung**

Singapore

**Houria Zait**

Algeria

**Zai-Xing Zhang**

China

**Hao-Bing Zhang**

China

**Long-Xian Zhang**

China

**Yi Zhang**

China

**Shao-Sen Zhang**

China

**Jin-Kou Zhao**

Switzerland

**Xiao-Nong Zhou**

China

**Xia Zhou**

China

**Yi-Biao Zhou**

China

**Shui-Sen Zhou**

China

**Denis Zofou**

Cameroon

**Guan-Yang Zou**

China

